# An Effective Color Quantization Method Using Octree-Based Self-Organizing Maps

**DOI:** 10.1155/2016/5302957

**Published:** 2016-01-14

**Authors:** Hyun Jun Park, Kwang Baek Kim, Eui-Young Cha

**Affiliations:** ^1^Department of Computer Engineering, Pusan National University, Busan 609-735, Republic of Korea; ^2^Department of Computer Engineering, Silla University, Busan 617-736, Republic of Korea

## Abstract

Color quantization is an essential technique in color image processing, which has been continuously researched. It is often used, in particular, as preprocessing for many applications. Self-Organizing Map (SOM) color quantization is one of the most effective methods. However, it is inefficient for obtaining accurate results when it performs quantization with too few colors. In this paper, we present a more effective color quantization algorithm that reduces the number of colors to a small number by using octree quantization. This generates more natural results with less difference from the original image. The proposed method is evaluated by comparing it with well-known quantization methods. The experimental results show that the proposed method is more effective than other methods when using a small number of colors to quantize the colors. Also, it takes only 71.73% of the processing time of the conventional SOM method.

## 1. Introduction

24-bit images can represent up to 16,777,216 colors. A variety of colors in an image provides the advantages of having greater abilities of expression and aesthetics. However, a variety of colors becomes a serious problem for most color image processing. Therefore, research to represent the various colors within a limited number of colors, which is called color quantization, has been conducted.

Self-Organizing Map (SOM), which is one of the most effective color quantization methods, provides excellent results [1–5]. However, low-frequency colors in the original image tend to be excluded during the learning process because of the characteristics of the SOM learning algorithm [6]. In addition, when SOM updates the winner node, it also updates neighbor nodes, which causes loss of the original color of the neighbor node. In particular, the loss of original color increases when SOM uses a small number of nodes to quantize the image with a small number of colors. Therefore, to complement these SOM disadvantages, we propose an effective color quantization algorithm that is effective even when it uses only a small number of colors by using an octree color quantization method.

Usually, mean absolute error (MAE), mean square error (MSE), and processing time are used to quantify the performance of the color quantization results. MAE and MSE are calculated by the following, respectively:

(1) MAEX,X^=1HW∑h=1H∑w=1WXh,w−X^h,w1,

(2) MSEX,X^=1HW∑h=1H∑w=1WXh,w−X^h,w22,

where *X* denotes the original image, $\hat{X}$ denotes the quantized image, and *H* and *W* denote image height and width, respectively.

## 2. Octree Color Quantization

An octree is one of the image-dependent methods classified as a hierarchical clustering method. It is faster than partitional clustering methods, but its performance is relatively poor [7, 8].

An octree is a tree structure with up to eight nodes as children. The octree can represent all colors in an image within an eight-level tree because the colors are represented with eight bits. At first, color distribution in the image is represented using an octree, which then prunes the nodes until *K* nodes remain. The palette colors are chosen from the remaining *K* nodes. This method is fast and gives good results [6, 9].

As mentioned above, octree color quantization generates the palette using the distribution of colors in the image, but it does not consider the frequency of color. This means that if an image is composed of similar colors overall but has many different low-frequency colors or noise, octree's results can be very poor.

**Figure 1:**
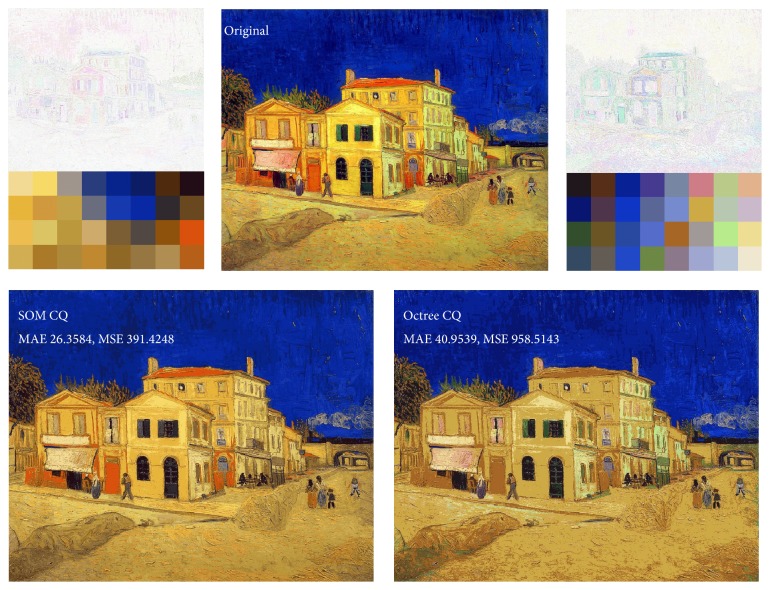
Strengths and weaknesses of octree color quantization (number of colors *K* = 32).

Figure 1 shows the strengths and weaknesses of octree color quantization. The original image has two high-frequency colors overall and many low-frequency colors around them. Yellowish and bluish colors occupy most of the image. Because SOM quantization considers the distribution of color, the generated palette has 21 yellowish colors and 5 bluish colors. By contrast, the generated palette from octree quantization has 5 yellowish colors and 9 bluish colors. Because of this, it is not influenced by the distribution of colors, and it creates a much greater difference from the original image. This is obviously octree's weakness.

However, octree has various colors, like greenish and pinkish, which SOM does not have. This is a strength for octree for the same reason.

In this paper, we present a more effective color quantization algorithm that complements the weakness of SOM by using the strengths of octree, which are fast and offer various colors.

## 3. SOM Color Quantization

SOM color quantization shows excellent results with most images. The results are very natural and have a low MAE and MSE because the SOM learning algorithm spontaneously considers the distribution of colors.

However, when SOM color quantization has only a small number of colors (i.e., the number of colors, *K*, becomes lower), its disadvantage is apparent. The SOM learning algorithm finds the winner node and updates its weight. At that time, neighbor nodes of the winner are also updated. This means SOM generates a more natural palette, but also it means SOM loses the original color of the neighbor nodes. High-frequency colors are learned many times, so they update the weights many times. This means that high-frequency colors influence neighbor nodes much more, because when the winner is updated, the neighbor nodes are also updated. It becomes more serious when the number of colors, *K*, becomes smaller. If *K* becomes too small, then the SOM map also becomes so small that the influences on updating the winner become much greater. Therefore, the overall colors can become similar and the low-frequency colors disappear through updating the weights.

**Figure 2:**
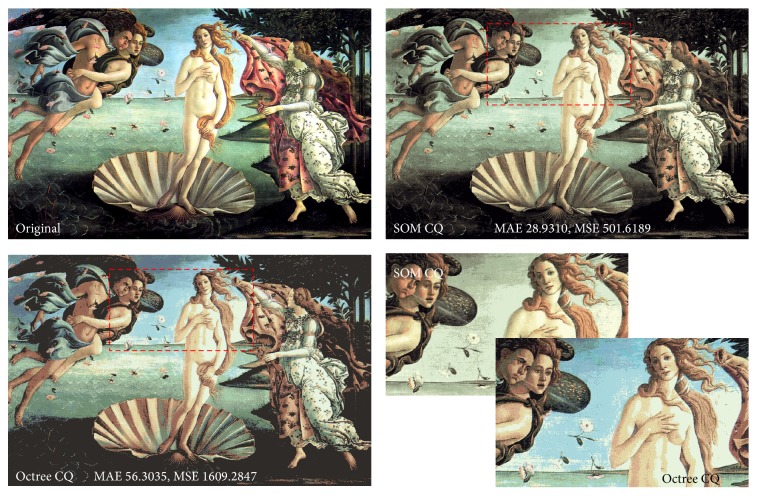
The disadvantage of SOM color quantization (number of colors *K* = 16).

Figure 2 shows quantized results with 16 colors from SOM [3, 6] and octree [9]. SOM (MAE 28.93, MSE 501.62) obviously has fewer differences from the original image than octree (MAE 56.30, MSE 1609.28). However, visually, the result of octree looks better because the results from SOM lost the sky-blue color, even if octree has almost double the MAE.

SOM color quantization is excellent method, but the number of colors, *K*, is small; then its performance is decreased. Therefore, it can be a problem when it is used to quantize an image with a small number of colors.

## 4. Octree-Based SOM Color Quantization

We present a more effective color quantization algorithm by using octree-based SOM color. It complements the disadvantages of SOM, so it not only gives visually natural results but also provides low MAE and MSE.

The beginning of the SOM learning algorithm initializes the weights with random values. It causes similar colors to be located anywhere on the palette. If they are high-frequency colors, then low-frequency colors around them are lost. Therefore, we intentionally initialize the weights with colors in the image. This can induce the results to be more similar to the original image with reduced loss of the original colors. Thus, because the initial weights are fixed, we can always get the same results, unlike traditional SOM.

The weight initialization should satisfy the following conditions to complement the problem above.

1. The time to create the map must be short.
2. The map should be created using the colors in the image.
3. It should be configured with a variety of colors from the image as much as possible.

Octree color quantization satisfies these conditions. It constructs the octree using the colors in the image, and it prunes them until only *K* nodes remain. It does not need much time to create the palette. In addition, it has various colors because it does not consider the distribution of color. Therefore, the proposed method initializes the weights using the palette generated by octree quantization.

**Figure 3:**
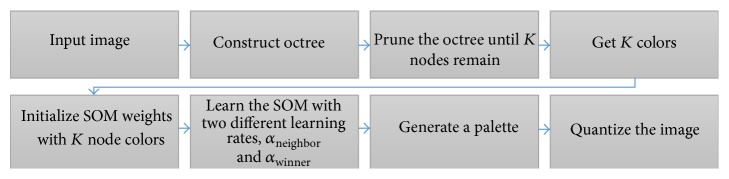
Overall process of octree-based SOM color quantization.

Figure 3 shows the overall process of the proposed method. At first, it builds an octree on the input image and prunes the octree until *K* nodes remain to get the *K* number of colors for initializing the weights of SOM [9].

After initializing, SOM learning is performed. The proposed method uses a two-dimensional SOM and two different learning rates for winner and neighbor nodes. The learning rate for neighbor node *α*_neighbor_ is a lot smaller than the learning rate for winner node *α*_winner_ in order to reduce the loss of low-frequency colors.

The proposed method uses different training data in each iteration. If SOM learns sequential pixels in an image, it means SOM learns similar pixels repeatedly because adjacent pixels in an image have similar colors. The training data for *t*th iteration, *d*_*t*_, is defined as follows for data sampling:

(3) $$\begin{array}{c} d_{t}=\left(\;x_{t},x_{2t},x_{3t},\ldots ,x_{N^{\prime }}\;\right). \end{array}$$

Determining the winner node and updating the weights are performed by traditional SOM learning method as shown in the following:

(4) winner=arg mink=1,…,K⁡dt,j−wk2,wnew=1−αwold+α·dt,j.

Algorithm 1 shows the learning algorithm of octree-based SOM color quantization.

**Figure 4:**
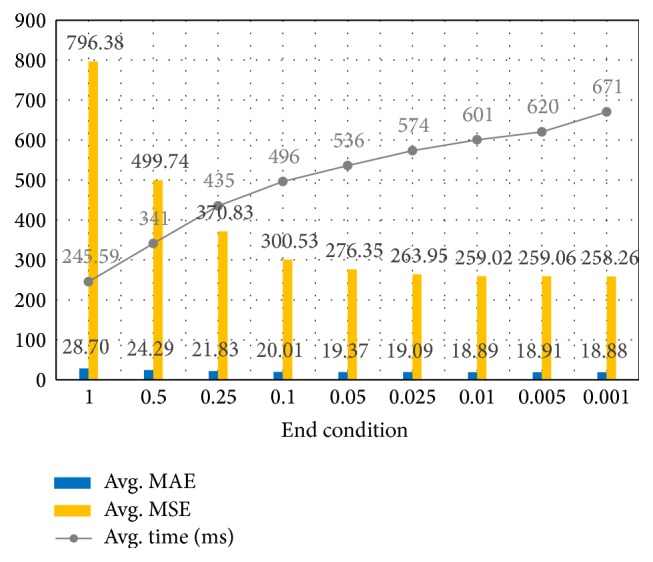
Average MAE, MSE, and processing time of all test cases.

In this paper, it assumes that the weights converge if the average variation in the weights is lower than 0.025, and it is used as an end condition. The end condition is experimental value.  Figure 4 shows average MAE, MSE, and processing time of all test cases. When the end condition is set between 0.01 and 0.025, it gives best performances. It shows the end condition 0.025 is reasonable.

## 5. Experimental Results

**Figure 5:**
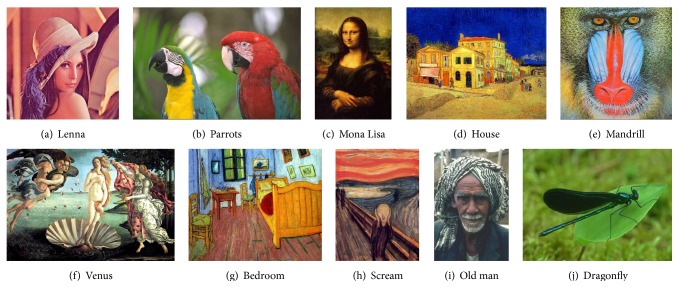
Test images.

The proposed method was tested on a set of ten true-color images commonly used to evaluate performance. The images are shown in Figure 5, and information about the test images is in Table 1. Test images (i) and (j) are collected from Berkeley color image database (http://www.eecs.berkeley.edu/Research/Projects/CS/vision/bsds). All of the color quantization methods were tested on an Intel i5-4460 3.2 GHz, 8.0 GB RAM machine and were implemented in C++.

The color differences shown in Table 1 were calculated with (5). As this value increases, the image has various colors, and the differences among the colors are greater:

(5) $$\begin{array}{c} \text{Color difference}=\frac{\sum_{i=1}^{N}\sum_{j=1}^{N}{\left\|x_{i}-x_{j}\right\|}^{2}}{{\left(N-1\right)}^{2}},\;\text{for }i\neq j. \end{array}$$

We evaluated the proposed method by comparing it with well-known color quantization algorithms, such as popularity (POP) [10], octree (OCT) [9], median-cut (MC) [10], *K*-means (KM) [11], Adaptive Resonance Theory 2 (ART2) [12], and Self-Organizing Maps (SOM) [3].  Table 2 explains these color quantization methods.

Tables 3 and 4 show a comparison of MAE and MSE, respectively. *K* means the number of colors used for quantizing an image. The best method is indicated in bold. As expected, the experimental results show that the proposed method gives better results than the conventional SOM method when *K* is small. It is the consequence obtained by reducing the learning rate of the neighbors. It minimizes the influence on the neighbor by the winner, so the proposed method has the original colors of the palette. It means the proposed method is more effective than other algorithms when *K* is small.

On the other hand, the disadvantage of SOM is reduced when SOM uses a big enough size of map for learning, because the influence of the winner is decreased. Therefore, the performance gap between conventional SOM and the proposed method also decreases when *K* is large. In some cases, the proposed method gives a larger MAE and MSE, because the low learning rate of the neighbor disturbs similar color grouping on the map.

Table 5 shows the processing time of the color quantization methods. The proposed method is faster than the conventional SOM method because the weights are initialized with the palette generated by octree. On the other hand, the conventional SOM method initializes the weights with random values. It makes SOM need less time before the weights converge. Therefore, the proposed method requires only 71.73% of the processing time of the conventional SOM method.

**Figure 6:**
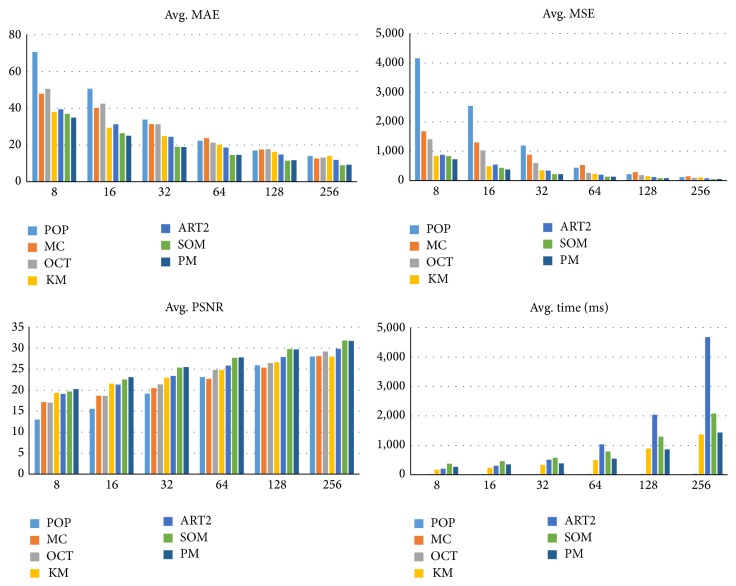
Average MAE, MSE, PSNR, and processing time of each method.

In Figure 6, the peak signal-to-noise ratio (PSNR) is additionally measured to evaluate quantization quality. PSNR is a popular measure to evaluate the reconstruction quality of image compression codecs and thus is used to evaluate color compression quality. A higher PSNR means the color quantization method has higher quality [13]. PSNR is defined as shown in the following:

(6) $$\begin{array}{c} \text{PSNR}=20\times \log_{10}\left(\;\frac{L}{\sqrt{\text{MSE}}}\;\right). \end{array}$$

**Figure 7:**
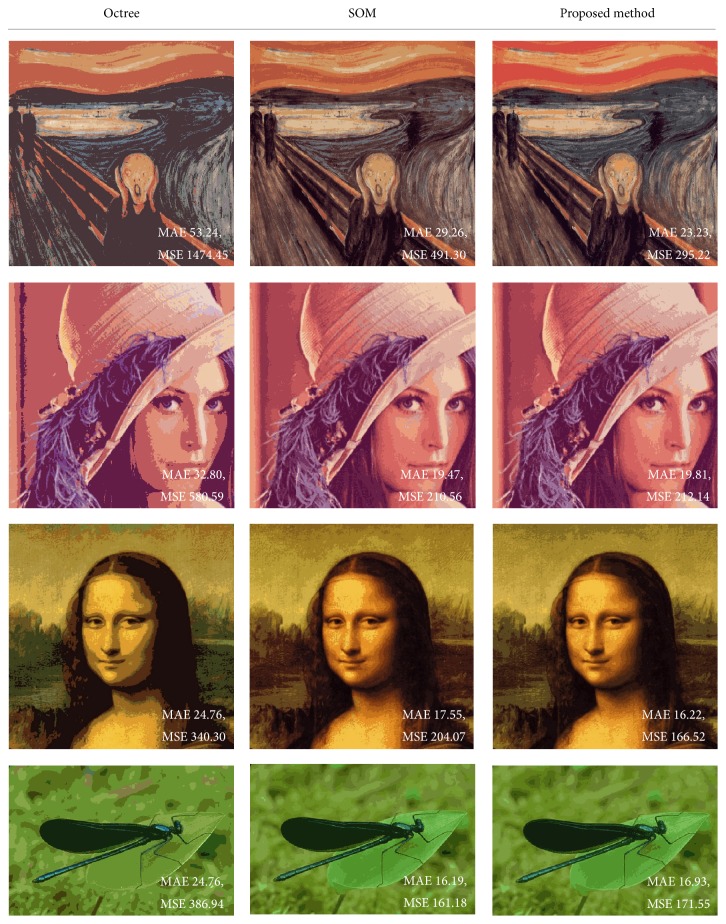
Resulting images from the color quantization methods (*K* = 16).

Figure 7 shows that the proposed method gives more similar results to the original image, visually, even if it has similar MAE and MSE. Overall colors in the palette generated by SOM become similar if *K* is small. However, the proposed method has colors more similar to the original image because of the low learning rate of neighbors and initialization by octree colors. The proposed method gives more similar sky color on scream image and gives more similar background color on Mona Lisa image.

## 6. Conclusions

In this paper, an effective color quantization method is proposed. Having many colors in the original image is a serious problem for image processing, so the number of colors must be reduced. Therefore, we propose an octree-based SOM color quantization method. It is particularly more effective than other algorithms when the number of colors, *K*, is small.

It uses an octree color quantization method to complement the disadvantages of SOM color quantization, which is one of the more effective methods. The octree color quantization method does not consider the distribution of colors, so its palette includes various colors. Therefore, we use the palette generated by octree to initialize the SOM weights. This causes the palette generated by SOM to have more varied colors than conventional SOM. In addition, the learning rate of the neighbors is set to low to minimize the loss of color information from updating the winner, because the influence of the winner becomes greater when *K* is small. The low learning rate of the neighbor means the proposed method can have colors similar to the original image.

The proposed method was evaluated by comparing it with six well-known quantization methods. MAE, MSE, and processing time are measured for comparison. The experimental results show that the proposed method is more effective than other methods when it uses a smaller number of colors to quantize the colors in the image (*K* = 8–32). It gives lower MAE and MSE. Thus, the proposed method requires only 71.73% of the processing time of the conventional SOM method. On the other hand, when it uses a large number of colors, the performance of the proposed method gives similar MAE and MSE with conventional SOM, but it is still faster.

It is expected that the proposed method will be available to many applications that have a problem with varied colors in images.

## Figures and Tables

**Algorithm 1:**
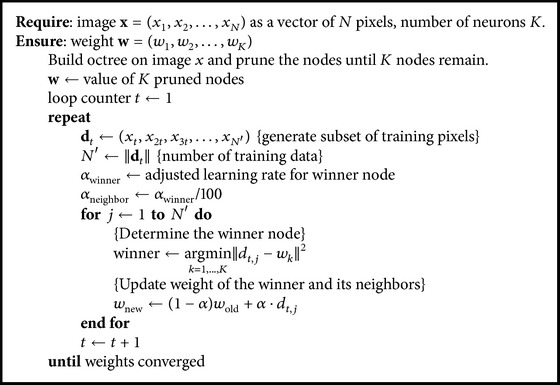
Learning algorithm of octree-based SOM color quantization.

**Table 1:** Information on test images.

Image	Resolution	Number of colors	Color difference
(a) Lenna	512 × 512	148,279	97.60
(b) Parrots	768 × 512	72,079	126.36
(c) Mona Lisa	1280 × 1920	125,240	97.14
(d) House	1280 × 1024	363,724	136.87
(e) Mandrill	512 × 512	230,427	118.48
(f) Venus	1848 × 1173	396,799	131.67
(g) Bedroom	1280 × 1024	454,673	136.26
(h) Scream	999 × 1362	189,162	108.71
(i) Old man	321 × 481	33,411	107.84
(j) Dragonfly	481 × 321	41,117	72.24

**Table 2:** Various color quantization methods [1].

Methods	Features of methods
POP [10]	(i) One of the simplest methods.
	(ii) 16 × 16 × 16 color histogram using 4 bits per channel uniform quantization.
	(iii) *K* most-frequent colors in the color histogram are used for quantization.

MC [10]	(i) 32 × 32 × 32 color histogram using 5 bits per channel uniform quantization.
	(ii) It makes cubes that include all of the histogram.
	(iii) It repeatedly splits the cubes that have the greatest number of colors until *K* cubes are obtained.

OCT [9]	(i) Tree structure with up to 8 nodes as children, which can represent all colors in an image within an 8-level tree.
	(ii) Color distribution is represented using octree, which then prunes the nodes until *K* nodes remain.

KM [11, 14, 15]	(i) It starts with *K* random clusters.
	(ii) All of the input data are assigned to the cluster that has the minimum distance within the data.
	(iii) The centroid of the cluster is calculated as the average of the assigned data.

ART2 [12]	(i) Unsupervised learning model.
	(ii) It creates new clusters depending on a vigilance test.
	(iii) The palette color is chosen from the centroids of the *K* most-frequent clusters.

SOM [1–5]	(i) Unsupervised learning model.
	(ii) One-dimensional self-organizing map with *K* neurons.
	(iii) It designates the minimum distance node as the “winner” node and then updates the weights of the winner node and neighbor nodes.
	(iv) It repeats the process until the sum of the weight change is less than a certain threshold.

**Table 3:** MAE comparison of the color quantization methods.

*K*	Lenna						Parrots					
	8	16	32	64	128	256	8	16	32	64	128	256
POP	41.5	36.6	22.0	16.1	12.7	11.9	101.6	73.5	58.8	22.5	16.5	13.6
MC	31.0	25.1	19.4	15.1	11.5	8.6	64.8	52.1	36.9	25.1	17.8	12.8
OCT	35.9	32.8	23.3	15.5	12.9	9.6	53.9	38.2	28.6	20.3	16.3	12.6
KM	31.1	30.1	24.8	19.7	13.3	12.0	44.7	31.0	26.6	20.2	16.6	12.9
ART2	31.1	22.1	18.5	12.4	10.3	8.5	43.8	35.3	25.9	18.3	14.2	11.3
SOM	27.4	**19.5**	**14.0**	**10.9**	**8.6**	**6.9**	42.4	28.4	20.3	**14.9**	**11.5**	**8.7**
PM	**27.1**	19.8	14.9	**10.9**	8.8	7.2	**41.0**	**27.8**	**20.1**	15.2	11.8	9.1

*K*	Mona Lisa						House					
	8	16	32	64	128	256	8	16	32	64	128	256

POP	40.4	28.1	20.4	14.2	12.9	12.5	62.6	52.2	39.1	29.7	21.5	15.8
MC	25.2	20.3	16.1	12.5	9.4	7.4	63.6	54.9	39.6	27.5	20.2	15.9
OCT	29.7	24.8	16.3	13.4	10.4	8.9	51.4	43.3	41.0	25.9	24.8	17.6
KM	30.7	23.5	18.8	14.2	13.6	11.8	43.1	30.8	25.7	22.1	18.5	16.0
ART2	29.2	21.8	16.3	12.3	9.5	7.6	44.1	33.4	29.3	23.6	18.8	15.3
SOM	25.9	17.6	13.3	9.7	7.3	**5.6**	47.3	33.2	**23.7**	**18.5**	**14.8**	**11.8**
PM	**24.0**	**16.2**	**12.0**	**9.1**	**7.2**	5.7	**42.0**	**30.6**	23.8	18.8	14.9	12.0

*K*	Mandrill						Venus					
	8	16	32	64	128	256	8	16	32	64	128	256

POP	78.6	56.9	40.9	31.0	21.1	15.7	109.6	46.5	33.0	23.8	17.3	13.8
MC	69.3	64.6	59.0	46.2	33.8	21.6	41.8	35.7	33.3	27.5	20.3	15.1
OCT	55.3	51.3	39.0	27.2	22.0	17.9	68.6	56.3	34.9	32.0	22.9	17.8
KM	46.3	34.9	30.3	22.5	18.7	16.8	43.5	34.3	25.5	20.8	17.6	14.7
ART2	50.7	37.3	29.2	24.8	20.0	16.3	44.6	37.4	31.2	22.3	17.9	13.7
SOM	45.3	**34.0**	**26.1**	**20.5**	**16.4**	**13.1**	42.5	30.3	21.9	16.9	**13.4**	**10.7**
PM	**45.0**	34.1	**26.1**	**20.5**	16.8	13.4	**40.9**	**28.4**	**21.2**	**16.7**	**13.4**	**10.7**

*K*	Bedroom						Scream					
	8	16	32	64	128	256	8	16	32	64	128	256

POP	102.1	96.5	56.0	36.1	27.1	19.0	59.3	47.3	29.5	20.9	14.6	12.3
MC	74.0	65.8	47.4	37.8	28.4	19.9	54.5	41.0	28.1	20.5	14.2	10.4
OCT	63.8	57.7	47.9	30.6	26.9	18.1	54.6	53.2	42.1	21.9	17.4	13.0
KM	53.8	40.2	33.0	25.9	21.0	17.3	33.6	27.8	24.2	21.7	15.2	13.0
ART2	52.6	47.5	35.6	27.8	21.7	17.7	33.0	26.3	19.8	15.3	12.5	10.3
SOM	53.8	39.2	28.4	21.8	**17.2**	**13.6**	40.5	29.3	18.0	**13.6**	**10.6**	**8.5**
PM	**51.7**	**37.3**	**28.0**	**21.7**	**17.2**	13.7	**33.1**	**23.2**	**17.6**	13.7	10.7	8.9

*K*	Old man						Dragonfly					
	8	16	32	64	128	256	8	16	32	64	128	256

POP	71.3	42.6	21.6	13.5	12.9	12.9	39.0	25.8	17.4	14.8	12.8	12.0
MC	27.9	19.0	15.2	11.1	8.8	6.8	26.9	22.7	18.1	13.8	10.9	8.3
OCT	51.6	40.2	17.7	12.0	9.4	7.0	40.2	26.9	22.4	14.3	13.6	9.0
KM	26.8	21.2	18.6	15.8	14.3	12.2	26.5	20.5	20.9	18.3	14.0	13.5
ART2	38.4	27.9	20.9	14.6	11.4	8.5	26.7	23.2	18.1	14.7	11.6	9.3
SOM	22.5	15.9	**11.4**	**8.7**	**6.5**	**5.2**	21.4	**16.2**	**12.7**	**10.0**	**8.9**	**6.2**
PM	**22.3**	**15.7**	11.8	**8.7**	6.8	5.5	**21.1**	16.9	13.4	10.6	9.6	6.9

**Table 4:** MSE comparison of the color quantization methods.

*K*	Lenna						Parrots					
	8	16	32	64	128	256	8	16	32	64	128	256
POP	1296.2	1144.6	336.1	175.1	79.8	64.8	8796.1	5976.1	4102.2	362.6	179.5	104.1
MC	577.8	412.4	264.5	163.6	94.8	47.0	2733.2	1716.3	999.7	508.6	274.4	142.9
OCT	692.6	580.6	273.5	126.1	85.4	47.5	1479.2	915.6	469.9	244.6	144.7	85.4
KM	515.3	476.0	334.2	204.9	96.4	77.5	1083.2	534.2	390.6	225.3	150.0	90.7
ART2	517.0	257.9	183.0	82.9	56.0	37.8	1085.8	657.3	353.4	179.7	105.8	68.0
SOM	400.6	**210.6**	**110.0**	67.1	**42.5**	**27.8**	1027.2	450.2	239.2	**133.0**	**78.8**	**46.8**
PM	**396.1**	212.1	121.5	**66.5**	43.1	28.9	**960.4**	**433.1**	**239.0**	135.6	81.0	47.9

*K*	Mona Lisa						House					
	8	16	32	64	128	256	8	16	32	64	128	256

POP	1330.1	658.7	320.2	103.4	76.5	69.0	2758.1	2044.4	1305.1	844.5	399.1	175.6
MC	496.5	330.9	211.7	131.2	60.3	33.2	2407.0	2003.1	1307.8	674.6	338.8	211.0
OCT	536.3	340.3	155.5	97.4	61.0	43.3	1451.2	1058.8	958.5	370.7	339.3	151.9
KM	597.4	339.1	203.0	117.8	103.5	76.4	1040.3	555.7	381.1	276.0	190.2	137.3
ART2	523.1	275.7	147.8	84.0	48.9	30.2	1137.8	620.0	481.0	306.3	189.7	121.6
SOM	483.0	204.1	118.2	60.4	33.7	20.5	1162.8	598.1	326.9	204.2	128.9	**81.7**
PM	**373.2**	**164.2**	**89.0**	**52.1**	**32.3**	**20.4**	**965.6**	**530.4**	**322.4**	**200.9**	**126.7**	**81.7**

*K*	Mandrill						Venus					
	8	16	32	64	128	256	8	16	32	64	128	256

POP	3878.9	2233.5	1200.6	740.0	305.7	151.7	7759.8	1591.6	899.7	450.8	208.9	110.4
MC	3308.9	3054.1	2614.9	1619.9	801.5	347.2	1107.6	884.4	805.7	577.9	303.0	179.2
OCT	1544.7	1358.6	841.2	379.1	245.6	153.7	2290.2	1609.3	600.3	497.9	286.2	150.6
KM	1137.8	647.2	486.3	266.8	183.4	147.6	1038.6	602.2	342.2	231.4	170.8	120.9
ART2	1339.1	733.4	437.0	319.6	206.3	135.4	987.4	691.0	499.0	261.7	166.0	99.8
SOM	1092.9	627.1	358.3	222.7	**144.1**	**93.1**	1067.0	577.3	292.5	173.5	107.3	68.5
PM	**1078.2**	**612.4**	**354.6**	**221.8**	149.7	94.0	**905.9**	**473.3**	**263.8**	**163.6**	**104.7**	**67.4**

*K*	Bedroom						Scream					
	8	16	32	64	128	256	8	16	32	64	128	256

POP	8107.1	7826.4	2374.7	1061.7	601.6	268.8	2609.4	1859.9	774.7	375.1	128.4	71.8
MC	3429.1	2859.7	1682.0	1121.3	659.6	314.7	1723.1	1149.2	501.3	277.6	133.1	72.8
OCT	2062.4	1753.1	1198.1	480.0	379.3	159.1	1528.1	1474.5	1054.3	256.5	170.2	85.8
KM	1554.1	868.5	588.0	361.6	232.8	156.9	613.1	404.6	312.0	246.7	124.1	88.7
ART2	1465.0	1152.9	647.0	400.9	245.9	158.8	581.6	356.3	204.9	121.3	80.7	54.2
SOM	1591.2	864.1	437.7	258.5	161.0	102.4	936.6	491.3	177.4	102.5	63.0	**40.3**
PM	**1433.4**	**750.0**	**423.3**	**253.0**	**160.0**	**101.4**	**592.1**	**295.2**	**166.6**	**101.8**	**62.0**	42.7

*K*	Old man						Dragonfly					
	8	16	32	64	128	256	8	16	32	64	128	256

POP	3643.5	1551.8	342.2	88.3	78.7	77.9	1427.9	512.6	230.4	157.5	97.4	66.4
MC	522.2	216.7	138.5	80.4	50.6	26.1	449.2	330.3	234.7	161.5	104.7	59.2
OCT	1432.0	764.2	178.3	75.5	46.1	25.4	990.4	386.9	264.2	105.8	95.2	40.8
KM	386.5	245.3	194.0	138.6	115.9	79.6	387.1	235.7	239.7	183.8	108.4	99.2
ART2	715.5	388.4	245.1	115.6	69.9	38.0	390.7	287.4	175.1	113.3	69.7	44.5
SOM	270.7	138.5	**75.4**	**41.1**	**24.9**	**15.8**	271.4	**161.2**	**98.9**	**60.8**	**43.8**	**24.3**
PM	**266.9**	**135.4**	78.5	41.2	25.1	16.3	**264.3**	171.6	103.1	63.2	50.1	26.6

**Table 5:** Processing time (ms) comparison of the color quantization methods.

*K*	Lenna						Parrots					
	8	16	32	64	128	256	8	16	32	64	128	256
POP	2	2	2	2	3	3	3	3	3	4	4	6
MC	3	2	2	2	2	3	3	3	4	3	4	4
OCT	6	6	7	7	8	8	9	11	10	12	12	13
KM	46	63	97	87	170	323	65	84	124	207	527	665
ART2	31	46	107	88	300	621	68	102	190	360	520	1001
SOM	93	107	139	213	333	548	148	160	207	311	471	825
PM	73	79	97	147	302	387	116	131	162	214	330	581

*K*	Mona Lisa						House					
	8	16	32	64	128	256	8	16	32	64	128	256

POP	21	19	28	23	21	23	11	11	11	11	12	16
MC	23	21	20	21	21	23	12	12	12	12	11	12
OCT	56	58	65	66	74	75	30	29	32	33	35	44
KM	321	646	778	1179	2246	2074	268	337	540	753	1150	2192
ART2	300	457	524	765	1462	6121	406	676	896	1930	3833	7722
SOM	863	938	1347	1678	2848	4621	485	681	754	1157	1791	2814
PM	612	708	896	1260	1979	3213	359	514	530	721	1135	1904

*K*	Mandrill						Venus					
	8	16	32	64	128	256	8	16	32	64	128	256

POP	2	2	3	3	3	5	18	18	20	20	19	22
MC	2	3	3	3	3	3	18	18	20	20	18	20
OCT	5	6	6	7	7	8	50	49	57	58	58	58
KM	46	66	96	134	230	441	455	487	740	1133	2065	3458
ART2	62	91	201	408	696	1428	631	760	1503	3408	6930	15597
SOM	108	130	156	220	467	593	953	985	1286	1839	3239	4898
PM	73	83	104	153	332	395	623	755	904	1246	1920	3325

*K*	Bedroom						Scream					
	8	16	32	64	128	256	8	16	32	64	128	256

POP	12	12	20	11	11	14	10	11	11	20	14	13
MC	10	13	12	11	13	13	13	18	13	13	12	12
OCT	28	28	30	31	33	66	32	31	34	38	37	42
KM	231	295	431	685	1159	2346	261	341	440	701	1183	1975
ART2	338	653	989	2183	4517	9675	179	210	624	1086	2026	4287
SOM	520	825	979	1103	1779	2944	477	688	740	1169	1700	3024
PM	349	549	515	726	1139	2063	413	633	525	841	1214	2054

*K*	Old man						Dragonfly					
	8	16	32	64	128	256	8	16	32	64	128	256

POP	1	1	1	1	2	2	3	1	1	2	2	2
MC	1	1	1	1	1	1	1	1	1	1	1	2
OCT	3	3	4	4	5	5	3	4	4	4	4	5
KM	19	26	41	39	56	72	19	27	44	68	119	163
ART2	14	16	19	29	43	76	16	19	27	40	72	259
SOM	37	46	78	100	155	257	37	47	64	100	162	277
PM	33	37	52	73	109	195	30	34	47	69	136	194
